# Age, Gender, and Liver Enzyme Impact Hospital Stay in COVID-19 Minority Patient with Cancer in the USA: Does Race Matters in the Pandemic?

**Published:** 2024-03-21

**Authors:** H Ashktorab, G Oskrochi, SR Challa, LG Chirumamilla, S Saroya, S Dusmatova, N Shayegh, V Nair, K Senthilvelan, D Byer, N Morrison, B Grossi, A Barclay, T Smith, K Watson, M Rashid, R Rashid, M Deverapalli, G Latella, JM Carethers, A Youssef, H Brim

**Affiliations:** 1 Department of Medicine, GI Division, Cancer Center, Howard University Hospital, Washington DC, USA.; 2 College of Engineering and Technology, American University of the Middle East, Kuwait.; 3 Gastroenterology Unit, Division of Gastroenterology, Hepatology, and Nutrition, Department of Life, Health and Environmental Sciences, University of L’Aquila, L’Aquila, Italy; 4 Division of Gastroenterology & Hepatology, Department of Medicine and Moores Cancer Center, UC San Diego, USA; 5 Division of Gastroenterology and Hepatology, Department of Internal Medicine; Department of Human Genetics and Rogel Cancer Center, University of Michigan, Ann Arbor, MI, USA; 6 Department of Pathology and Cancer Center, Department of Biochemistry & Molecular Biology, Howard University College of Medicine, Washington DC, USA

**Keywords:** Coronavirus (COVID-19), Cancer, African American, Mortality

## Abstract

Patients with cancer are known to have a poor prognosis when infected with SARS-CoV-2 infection. We aimed in this study to assess health outcomes in COVID-19 patients with different cancers in comparison to non-cancer COVID-19 patients from different centers in the United States (US). We evaluated medical records of 1,943 COVID-19 Cancer patients from 3 hospitals admitted between December 2019 to October 2021 and compared them with non-cancer COVID-19 patients. Among 1,943 hospitalized COVID-19 patients, 18.7% (n=364) have an active or previous history of cancer. Among these 364 cancer patients, 222 were African Americans (61.7%) and 121 were Caucasians (33.2%). Cancer patients had significantly longer hospitalization compared to controls (8.24 vs 6.7 days). Overall, Lung cancer is associated with high mortality. Patients with a previous history of cancer were more prone to death (p=0.04) than active cancer patients. In univariate and multivariate analyses, predictors of death among cancer patients were male sex, older age, presence of dyspnea, elevated troponin, elevated AST (0.001) and ALT (0.05), low albumin (p=0.04) and mechanical ventilation (p=0.001). Patients with a previous history of cancer were more prone to death when compared to active cancer COVID-19 patients. Early recognition of cancer COVID-19 patients’ death-associated risk factors can help determine appropriate treatment and management plans for better prognosis and outcome.

## 2. Introduction

Background: COVID-19 pandemic accounts for more than 6,181,850 deaths globally, with 978,118 in the USA until April 2022 [1]. COVID-19 patients with pre-existing conditions are at increased risk of poor clinical outcomes. More specifically, cancer patients are at a high risk of developing serious outcomes such as intensive care unit (ICU) admission, mechanical ventilation, and/or death [2–4]. Reports from China show that the case fatality rate of cancer patients with COVID-19 is approximately double that of other COVID-19 patients (5.6% vs. 2.3%) [4, 5]. In an EHR-based study (Electronic Health Record) of 1,200 cancer patients, African Americans with cancer had a significantly higher risk of COVID-19 when compared to Caucasians [6]. Data have suggested that cancer is a significant risk factor for mortality from severe COVID-19 with increased risk for ICU admission and mechanical ventilation [6, 7]. Thus, further research regarding the diagnosis and management is recommended to protect such vulnerable populations. Cancer patients are more susceptible to infections because of their systemic immunosuppressive state caused by the malignancy combined with the anti-cancer treatments [6, 8, 9]. Recent studies have shown that lung, blood, and all metastatic cancers worsen the clinical course of COVID-19 [4, 6]. A prospective cohort study with COVID-19 patients showed that those who had recently undergone cancer treatment had a higher risk of severe outcome than untreated cancer patients [4]. Several studies have attempted to define prognostic factors that could assist in risk stratification and clinical management [8]. Therefore, in addition to reducing exposure to the virus, other prophylactic and cancer-related risk factors may need to be addressed to decrease susceptibility to COVID-19 or mitigate related complications in cancer patients

## 3. Objective

We aim to perform a comprehensive analysis on patients with COVID-19 from 3 US tertiary care hospitals addressing outcomes, clinical characteristics, demographic profiles, and ICU admission rates. Also, we focused on COVID-19 patients with cancer (Cases) to determine whether they have a different prognosis compared to non-cancer COVID-19 patients (Controls).

## 4. Methods

### 4.1. Study Design and Setting

This is a retrospective case-control study on hospitalized patients with COVID-19. This study included patients from 3 different hospitals in the USA, Howard University Hospital (DC), SUNY Downstate Medical Center (New York), and Henry Ford Hospital (Michigan). We analyzed de-identified hospitalized patients’ data, using the EHR from each hospital between Dec. 2019 to Oct. 2021. This study was approved by Howard University Institutional Review Board (IRB). An excel file template was shared with our collaborators for data collection.

### 4.2. Study Participants

Inclusion criteria: All patients were screened for cancer based on the presence of a corresponding ICD-10 code (International Classification of Diseases, Tenth Revision) in their EHR. The following criteria were adopted to validate patient selection: patients with a confirmed diagnosis of COVID-19 (PCR positive) with a specified past history or current diagnosis of any cancer as Cases. Patients without cancer but with a confirmed diagnosis of COVID-19 were included as Controls. Exclusion criteria: Were excluded: Patients with negative SARS-CoV-2 PCR; Patients below 18 years of age. Patients without past history or current diagnosis of cancer were excluded from the Case group.

### 4.3. Sample Size and Variables

A total of 1943 COVID-19 positive patient’s data was received from all 3 hospitals, by reviewing their EHR during the specified time. We compared patients baseline demographics, comorbidities, clinical manifestations, laboratory parameters, ICU admission, mechanical ventilation, hospital length of stay and death. Patients with diagnosis of cancer within 4 weeks of admission was considered as active and more than 4 weeks before admission was considered as previous history of cancer. Type of cancer was determined based on the organ specific location. Total number for each variable in the overall analysis varies due to some missing values.

### 4.4. Statistical Analysis

Data was collected in an encrypted excel sheet and the frequency of each variable was compared between cases and controls, using computed Demographics, clinicals, and outcomes were compared among cases and controls. Variables included age, gender, race, comorbidities, clinical manifestations, ventilation, D-dimer, ferritin, troponin, alanine aminotransferase (ALT), aspartate transaminase (AST), lactate dehydrogenase (LDH) levels, ICU admission and hospital stay duration. Data was analyzed using SPSS software. We performed univariate (univariate logistic regression, chisquared and T-test) and multivariate analysis (multivariable logistic regression) with a statistical significance of P-value <0.05. In the multivariable analysis, important and appropriate risk factors and demographic factors were included as control in each analysis. Missing values were regarded completely missing at random as there was no indication that missing information is related to outcome under investigation.

## 5. Results

### 5.1. Demographics of the COVID-19 Cancer Patients vs. Non-Cancer Hospitalized Patients

Our study collected data from 1,943 hospitalized COVID-19 patients. Among them, 364 (18.7%) patients have an active or history of cancer. Baseline characteristics of patients (cases and controls) time of COVID-19 diagnosis are shown in (Table 1). Patients with cancer are older when compared to the non-cancer COVID-19 patients (69.7 vs. 61.3 years; p<0.001) and comparatively less likely to be female in both cases (48.4% vs. 51.6%) and controls (48.3% vs. 51.7%) (Table 1). The majority of the patients were African Americans in both cases (n=222, 60.9%) and controls (n=900, 57%). Obesity was prevalent in both cases (37%) and controls (44.9%). Mortality rate was higher among the cases (26.1%) compared to controls (23.1%).

### 5.2. Comorbidities, Clinical Manifestations, and Labs of Cancer Patients with COVID-19 Compared with Controls

Significant comorbidities that are more common in cancer patients with COVID-19 (cases) compared to non-cancer COVID-19 patients (controls) are hypertension (n=278, 76.4%), congestive heart failure (n=72, 22.6%), renal disease (n=130, 35.7%) and COPD (n=66, 18.1%) (Table 1). Fever (n=62, 19.5%), cough (n=84, 23.1%) and dyspnea (n=113, 35.4%) are the most common significant symptoms in cases compared to controls (Table 1). Among laboratory tests, elevated BUN, elevated creatinine, and elevated glucose are significantly more common in cases when compared with controls (Table 1).

### 5.3. Most Common Cancer Types and Treatment in Cancer Patients infected with COVID-19

Among the 364 COVID-19 patients with an active or history of cancer, the most common cancer in female patients is breast (41.5%), followed by Head and Neck cancers (11.4%) (Table 2). In males, prostate cancer (41.5%) followed by Hematological cancers (10.2%) were most prevalent (Table 2). However, mortality is high among lung cancer patients exposed to COVID-19 (n=12, 38.7%) (Table 2). Of the total, 92 (25.3%) cancer patients with COVID-19 had received active cancer treatment within the preceding 4 weeks, and 170 (46.7%) received cancer treatment in less than six months prior to infection. Chemotherapy (n=120, 33%) was the most common cancer treatment received by the cases in this study (Table 2). Neither the type of anti-cancer treatment nor the treatment time is associated with mortality in cancer patients with COVID-19 in this study.

### 5.4. Predictors of Mortality in Cancer Patients Exposed to COVID-19 vs. Non-Cancer COVID-19 Patients’

Male sex (p=0.002) and older age (p=0.001) are more prone to death in both cases and controls (Table 3 and 4). There is no effect of race/ethnicity on death among cases whereas, among controls, African American race is associated with high mortality. Cases did not show any effect on the correlation between clinical comorbidities and death. Presence of dyspnea (35.4%, p=0.004) at the time of admission was associated with poor outcomes. In contrast, comorbidities such as congestive heart failure (CHF), coronary artery disease (CAD), COPD and fever are associated with poor outcomes in controls (Table 4). Elevated troponin (p=0.003), AST (0.002), and decrease in albumin (p=0.02) are associated with high mortality among cases (Table 3), whereas elevated Ferritin (p=0.05), glucose (0.009), troponin (p=0.001) and BUN (p=0.03) are more prone to worse outcomes among controls (Table 4). ALT is also associated with mortality in cases at the 10% level (p=0.057). Compared to active cancer patients, past history of cancer (p=0.04) was associated with high mortality (Table 3). Mechanical ventilation is associated with poor outcomes in both cases and controls (Table 3 and 4).

## 6. Discussion

COVID-19 has impacted the whole world in all facets. Patients with chronic illnesses, including cancer, were adversely affected during this pandemic. The risk of infection and poor response is generally viewed as high in immunocompromised patients, including those with cancer or under cancer treatment [10]. Among 1,934 hospitalized COVID-19 patients, there were 364 cancer patients. The average age of the patients was 69.7 years. The presence of cancer apparently accentuates the increased risk of COVID-19 severity identified for men and the elderly [11]. Older age and male sex are associated with adverse outcomes in this study. Previous studies have identified that African American cancer patients were at significantly increased risk for COVID-19 infection and worse outcomes [6]. This may be due to the observation that irrespective of race/ethnicity, cancer may predispose any patient to SARS-CoV-2 infection as well as COVID-19 severity, whereas in those without cancer, certain populations such as African Americans have other comorbidities over Caucasians that place them at higher risk for COVID-19 [11]. In our study, African American COVID-19 patients without cancer have high mortality compared to other ethnicities. Our results showed that the most common cancer types were breast and prostate cancer among females and males, respectively. Irrespective of sex, hematological malignancies were more common overall in this study. Lung cancer was associated with the highest mortality among COVID-19 patients. Previous studies in the US have shown that hematological cancers are associated with high mortality compared to other cancer-COVID-positive patients [6, 8, 12]. A recent study showed that patients with lung and hematologic cancer display higher susceptibility of severe events in proportion to high viral load. Higher mortality and severity were directly proportional to high viral load [13].

In our study, previous history of cancer is associated with high mortality among COVID-19 patients. In several other studies, patients with active cancer are more prone to death than those with a history of cancer [6, 8, 14]. This difference could be due to cancer treatments such as chemotherapy, and radiation therapy that can suppress or weaken the immune system. Superimposed COVID-19 infection in such patients can further aggravate the condition leading to worse outcomes. The exact timeline of these therapies with the exposure and infection with SARS-CoV-2 is detrimental to know. The potential correlation with outcome. A practical aspect of this finding is to know when best to administer vaccines in cancer patients for better immune response. Indeed, Herishanu et al. showed that in patients with Chronic Lymphocytic Leukemia (CLL), the proper and efficacious immune response to Pfizer vaccine (and subsequently to infection) depended on disease activity and treatments [15]. So far, studies have shown that patients with cancer were at significantly increased risk for COVID-19 infection and worse outcomes [6, 14], but in our study, even though the percentage of deaths and ICU admission among the cases is high when compared to controls, this was not significant. Mortality of hospitalized cancer patients in our study is high (26.1%), similar to other recently published articles [14, 16–18]. However, this increased mortality in hospitalized cancer patients with COVID was not significant compared to the controls. Similarly, a study conducted in New York found no significant difference in relative risk of death in patients with cancer [19].

Typical symptoms of COVID-19 in cancer patients in this study (fever, cough, dyspnea, and fatigue) did not differ from those reported in the general population with COVID-19 [20, 21]. In this study, dyspnea was the only significant symptom (p<0.004) associated with high mortality. Barros et al. and Acar et al. have reported similar results for dyspnea in their study [9, 22]. This finding suggests that shortness of breath should be given special attention in managing hospitalized cancer patients with COVID-19. Cancer patients with pre-existing comorbidities such as hypertension, congestive heart failure, chronic kidney disease, and COPD are significantly more likely to get infected with COVID-19 than controls [11]. Among laboratory test parameters, D-dimer, troponin and AST were significantly elevated in cases compared to control patients. Predictors of poor outcomes among cases in this study are elevated troponin, AST, and decreased albumin. Low albumin levels predicted worse outcomes in cancer with COVID-19 in several other studies [22–24]. So, clinicians should give special attention to patients with low albumin. Our study has some limitations. The number of cancer patients was small and did not report relevant data such as cancer staging, presence of metastasis and ECOG performance status. Secondly, we analyzed only hospitalized patients, so we do not have information on outpatients. The findings of our study reflect the outcomes of adult patients treated in the US, and the outcomes may differ in settings with fewer resources or in areas of the world with limited health care capacity. Our findings can’t be generalized because our study was retrospective and involved only 3 centers in the US.

## 7. Conclusion

Active preventive care measures should be taken care in the immunosuppressed patients to prevent hospitalization. Early recognition of risk factors and addressing the laboratory parameters, accounting for poor prognosis is strongly recommended. There is a need to evaluate the role and efficacy of COVID-19 vaccines and treatment with anti-viral in cancer patients. More extensive studies are recommended on COVID-19 infection in cancer patients.

## Figures and Tables

**Table 1:** Demographics and clinical characteristics of hospitalized COVID-19 patients with and without cancer.

Demographics and clinical characteristics	Covid-19 Patients with active or past history of Cancer (n=364)	Covid-19 patients without active/past history of Cancer (n=1579) n (%)	p-value
Age (Mean, Years)	69.75	61.34	< 0.001
Sex	n (%)	n (%)	
Male	188 (51.6)	816 (51.7)	
Female	176 (48.4)	762 (48.3)	
Race			
African American	222 (60.9)	900 (57)	<0.001
White	121 (33.2)	408 (25.8)	
Hispanic	1 (0.2)	100 (6.3)	
Asian	9 (2.5)	99 (6.2)	
Others/Unknown	11 (3.02)	72 (4.5)	
Length of the Hospital stay (Mean days)	8.2	6.7	0.02
BMI			
Normal	98 (27.7)	207 (21.5)	0.53
Underweight	11 (3.11)	25 (2.6)	
Overweight	113 (32)	297 (30.9)	
Obese	131 (37)	431 (44.9)	
Comorbidities			
Hypertension	278 (76.4)	590 (37.4)	< 0.001
Diabetes Mellitus	144 (39.6)	584 (37)	0.447
COPD	66 (18.1)	85 (6.9)	< 0.001
Asthma	73 (20.1)	498 (31.5)	< 0.05
CHF	72 (22.6)	77 (10.4)	< 0.001
CAD	95 (26.1)	333 (21.4)	> 0.05
Renal	130 (35.7)	296 (24.5)	< 0.001
Symptoms			
Shortness of breath	113 (35.4)	403 (25.5)	<0.001
Fever	62 (19.5)	285 (26.4)	0.013
Cough	84 (23.1)	521 (33.3)	<0.001
Chest Pain	3 (1.1)	15 (2)	0.331
Myalgia	12 (3.8)	135 (18.8)	<0.001
Fatigue	29 (9.1)	133 (13.8)	0.03
Abdominal Pain	7 (2)	61 (5.8)	0.01
Diarrhea	13 (3.8)	129 (9.6)	0.01
Vomiting	7 (2)	109 (8.1)	0.001
ICU Admission	73 (23)	160 (14.7)	
Mechanical Ventilation	55 (15.1)	261 (16.5)	
Mortality	95 (26.1)	364 (23)	
Laboratory parameters			
Ferritin	184 (74.5)	592 (70.4)	0.187
D-Dimer	221 (91.3)	612 (83.6)	0.003
Troponin	131 (48.7)	259 (40.8)	<0.03
BUN	144 (42.6)	359 (47.6)	<0.001
Creatinine	151 (47.6)	371 (34)	<0.001
Glucose	90 (33.3)	370 (49.1)	<0.001
Bilirubin	45 (16.2)	141 (14.2)	0.397
AST	141 (54.7)	502 (51)	<0.001
ALT	35 (12.9)	142 (15.1)	0.231
Death	95 (26.1)	364 (23.05)	0.217

**Table 2:** Clinical characteristics of cancer patients exposed to COVID-19 (Cases)

Clinical Characteristics	Alive	Dead
1. Type of Cancer (n=364)	N=269	N=95
Head and Neck Cancer	34 (12.64%)	5 (5.26%)
Renal Cancer	17 (6.32%)	8 (8.42%)
Hematological Cancer	26 (9.67%)	8 (8.42%)
Skin Cancer	10 (3.72%)	5 (5.26%)
Lung Cancer	19 (7.06 %)	13 (13.68%)
Gynecologic Cancer	13 (4.83%)	5 (5.26%)
Colon Cancer	19 (7.06%)	6 (6.32%)
Prostate Cancer	50 (18.59%)	30 (31.58%)
Breast Cancer	60 (22.30%)	15 (15.79 %)
Liver Cancer	6 (2.23%)	2 (2.11%)
Other GI	9 (3.35%)	4 (4.21%)
Unknown	7 (2.60%)	1 (1.05%)
2. Cancer Status (n=316)	N=251	N =65
Active	67 (26.69%)	10(15.38%)
Previous History of Cancer	157 (62.55%)	54 (83.08%)
Both (Active and Previous history)	27 (10.76%)	1 (1.54%)
3. Status of Anti-Cancer Treatments (n=278)	N=221	N=57
Active (within last 4 weeks)	78 (35.3%)	14 (24.6%)
Greater than 4 weeks and less than 6 months	129 (58.4%)	41 (71.9%)
Greater than 6 months	14 (6.3%)	2 (3.5%)
4. Type of Anti-Cancer treatment (n=263)	N=206	N=57
Chemotherapy	98 (47.57%)	24 (42.11%)
Radiotherapy	98 (47.57%)	26 (45.61%)
Surgery	74 (35.92%)	19 (33.33%)
Immunotherapy	7 (3.40%)	2 (3.51%)
Hormonal Therapy	22 (10.68%)	5 (8.77%)
5. Metastasis (n=50)	N = 38	N=11
Yes	6 (15.79%)	4 (36.4%)
No	32 (84.21%)	7 (63.6%)

**Table 3:** Predictors of Mortality in hospitalized Cancer patients exposed to Covid-19

Predictors	n (%)	p-value
Age	-	0.002
Male (n=364)	188 (51.6%)	0.001
Previous history of Cancer (n=269)	157 (49.7%)	0.04
Laboratory Parameters		
Troponin (> 0.04 ng/ml) (n=269)	131 (48.7%)	0.001
AST (> 48 U/L) (n =258)	141 (54.6%)	0.001
Albumin (< 3.4 g/dl) (n=68)	28 (43.8 %)	0.04
Mechanical Ventilation (n=361)	55 (15.2 %)	0.001

**Table 4:** Predictors of mortality in COVID-19 patients without cancer

Predictors	Total n (%)	p-value
Age	-	0.0001
Male sex	816 (51.7%)	0.01
African American Race	900 (57%)	0.0001
Comorbidities:		
Coronary artery disease	333 (214)	0.001
Congestive heart failure	77 (10.4%)	0.001
COPD	85 (6.9%)	0.05
Labs:		
Ferritin	592	0.05
Troponin	259	0.001
BUN	359	0.03
Glucose	370	0.009
Mechanical Ventilation	261 (16.1%)	0.0001

## Data Availability

The data generated in this study are available upon request from the corresponding author.
